# Intraoperative methadone in adult laparoscopic surgery: a systematic review and meta-analysis of randomized trials

**DOI:** 10.1016/j.bjane.2026.844848

**Published:** 2026-10-15

**Authors:** Julio S. Curi, Matheus Arraes, Jasmeen Kaur, Carlos Martins, Rafael A. Neves, Eduardo M. Pereira, Shaun E. Gruenbaum, Rafael A. Lombardi

**Affiliations:** a Universidade do Vale do Taquari, Department of Medicine, Lajeado, RS, Brazil; b CMA Anesthesia Group, Division of Anesthesiology, São Paulo, SP, Brazil; c Augusta University Medical Center, Department of Anesthesiology, Georgia, USA; d Grupo de Anestesia Anestesvel, Department of Anesthesiology, Cascavel, PR, Brazil; e Centro Universitário Fundação Assis Gurgacz, Department of Medicine, Cascavel, PR, Brazil; f Universidade Federal de Minas Gerais, Department of Medicine, Belo Horizonte, MG, Brazil; g Mayo Clinic Florida, Department of Anesthesiology, Florida, USA; h University of Nebraska Medical Center, Department of Anesthesiology, Nebraska, USA

**Keywords:** Analgesia, Analgesics, opioid, Anesthesia recovery period, Laparoscopy, Methadone, Pain, postoperative

## Abstract

**Background:**

Opioids are routinely used for pain control in laparoscopic procedures. Methadone, a long-acting opioid, has unclear efficacy in this context. Therefore, we aim to compare intraoperative methadone *vs*. short-acting opioids or placebo for laparoscopic surgeries.

**Methods:**

We searched PubMed, Scopus, Embase, and Cochrane for eligible Randomized Controlled Trials (RCTs) up to June 2025. Outcomes were cumulative opioid use at 24 hours (primary endpoint), the incidence of respiratory depression, postoperative nausea/vomiting, and hypoxia; intraoperative propofol use; emergence time; Post-Anesthesia Care Unit (PACU) length of stay and opioid use; pain scores at 30 and 60 minutes and at 24 hours (h). We used a random-effects model, Cochrane risk-of-bias analyses, certainty-of-evidence assessment, trial sequential analyses, and minimal clinically important differences to assess clinical impact.

**Results:**

7 RCTs (591 patients) were included. Compared to short-acting opioids, methadone was significantly associated with a reduction in postoperative hypoxia (RR = 0.24; 95% CI 0.06–0.91; p = 0.03), PACU opioid use (RR = 0.69; 95% CI 0.53–0.90; p < 0.01), pain scores at 30 (MD = -1.21; 95% CI -1.91 to -0.51; p < 0.01) and at 60 minutes (MD = -0.41; 95% CI -0.80 to -0.03; p = 0.03), and cumulative opioid use at 24h (MD = -1.62; 95% CI -2.75 to -0.50; p < 0.01). There were no differences between groups in the remaining outcomes.

**Conclusion:**

Intraoperative methadone in laparoscopic procedures was associated with a statistically significant reduction in pain scores, hypoxia, and opioid requirements. However, the reduction in opioid requirements did not reach clinical significance, and most effects were small, heterogeneous, and supported by limited-certainty evidence. Evidence regarding respiratory adverse events remains limited because of the small number of trials and participants.

## Introduction

Postoperative pain remains a major challenge in laparoscopic procedures. This issue is especially significant in outpatient surgeries, which are increasingly common in modern clinical practice, where quick recovery is crucial for early hospital discharge. Poor pain management in surgical patients is linked to higher morbidity, slower recovery and discharge, reduced quality of life, prolonged opioid use, and increased health care costs.^1^, ^2^, ^3^

Methadone is a long-acting μ-opioid receptor agonist;^4^ nevertheless, it also acts as a non-competitive antagonist of N-Methyl-D-Aspartate (NMDA) receptors and inhibits serotonin and norepinephrine reuptake in the central nervous system.^5^ It has a half-life ranging from 13 to 50 hours.^6^^,^^7^ Because of its long-acting nature, it provides a stable plasma concentration after a single intraoperative dose when compared to fluctuating levels seen with short-acting opioids like fentanyl, morphine, or hydromorphone.^6^^,^^8^^,^^9^

Some studies have shown positive results when using intraoperative methadone in spine, cardiac, pediatric, and general surgery for managing acute postoperative pain and decreasing opioid requirement.^10^, ^11^, ^12^, ^13^, ^14^ Similarly, several Randomized Controlled Trials (RCTs) and observational studies have investigated the use of methadone in laparoscopic surgeries and have shown reduced opioid requirement in bariatric, gynecological, ambulatory, and cholecystectomy surgeries.^15^, ^16^, ^17^, ^18^, ^19^ However, these studies are limited by small sample sizes and have yielded inconsistent outcomes. Additionally, few studies have assessed metrics such as quality of recovery or functional outcomes in the laparoscopic setting.

We, therefore, aim to conduct a systematic review, meta-analysis, and Trial Sequential Analysis (TSA) of RCTs to study the effectiveness of methadone *vs*. standard opioid therapy in the management of acute postoperative pain.

## Methods

This study follows the Preferred Reporting Items for Systematic Reviews and Meta-Analyses (PRISMA) guidelines.^20^^,^^21^ The protocol was preregistered using the International Prospective Register of Systematic Reviews (PROSPERO, CRD420251075236).

### Eligibility criteria

The inclusion criteria were defined as 1) RCTs; 2) Comparing methadone *vs*. no methadone or placebo intraoperatively; 3) Including patients ≥ 18 years old undergoing laparoscopic surgeries; and 4) Reporting at least one outcome of interest. We excluded 1) RCTs with overlapping populations; 2) Conference abstracts; and 3) Studies using regional anesthesia for all patients. We did not apply restrictions on language or publication year. We contacted the authors of individual RCTs to obtain missing data. We excluded studies using any regional anesthesia (i.e., neuraxial anesthesia or peripheral blocks, excluding port-site infiltration) for all patients.

### Search strategy and data extraction

Two independent authors (R.L. and C.E.) systematically searched MEDLINE, Embase, Scopus, and Cochrane databases for RCTs meeting the inclusion criteria from inception until June 2025. The search was updated through October 1, 2026, and no additional eligible randomized trials were identified. The search strategy is listed in Supplementary Table 1. Additionally, we reviewed references from all included studies to identify additional relevant studies (backward snowballing). Nonetheless, we did not search the grey literature for additional studies. Three independent authors (R.L., C.E., and J.C.) collected the data and resolved disagreements through consensus. As of August 2026, each reference was checked to ensure that no cited study underwent retraction, withdrawal, expression of concern, or correction in the source database or publisher's website.

### Endpoints

The included outcomes of interest were 1) Cumulative postoperative opioid consumption within the first 24 hours after surgery, expressed as Oral Morphine Equivalents (OME); 2) Emergence time; 3) Incidence of hypoxia; 4) Incidence of PACU opioid consumption; 5) Incidence of Postoperative Nausea and Vomiting (PONV), defined as any episode of nausea, vomiting, or both during the postoperative observation period; 6) Incidence of respiratory depression, defined as a respiratory rate < 10 breaths per minute sustained for at least 10 minutes; 7) Intraoperative opioid consumption expressed in OME; 8) Intraoperative propofol consumption; 9) PACU length of stay; 10) PACU (0-min) pain scores; 11) 30 minutes pain scores; 12) 60 minutes pain scores; 13) 24 hours pain scores; and 14) Patient satisfaction. The primary outcome was cumulative 24h postoperative opioid use; all the other outcomes listed above were our secondary outcomes. We assessed pain outcomes using a numeric rating scale (NRS 0 to 10). When studies reported pain using the Visual Analog Scale (VAS 0–100 mm), we converted to NRS by dividing by 10, as NRS and VAS scores are considered sufficiently comparable to permit pooling on the same scale while preserving clinical interpretability.^22^ Opioid doses were converted to OME using the ClinCalc opioid conversion calculator.^23^ We pooled results for additional outcomes whenever they were reported in at least three studies. We extracted and analyzed hypoxia according to each trial’s original definition. Freire et al.^16^ defined hypoxia as sustained desaturation or hypoventilation associated with peripheral oxygen saturation below 90%, whereas Pontes et al.^15^ defined it as peripheral oxygen saturation of 90% or lower. Moro et al.^19^ reported hypoxemia without providing an operational definition. No standardized oxygen-saturation threshold was applied retrospectively.

### Quality assessment

Risk of bias was assessed with version 2 of the Cochrane Risk of Bias assessment tool (RoB2) by two independent authors (R.L. and J.C.).^24^ Furthermore, two authors (R.L. and M.A.) independently applied the Grading Recommendations, Assessment, Development, and Evaluation (GRADE) tool to assess the certainty of evidence for each outcome. Similarly, we resolved any disagreements through discussion with the remaining authors. We also performed a leave-one-out sensitivity analysis for each outcome.

### Statistical analysis

We pooled dichotomous outcomes using the Mantel-Haenszel method and reported Relative Risks (RRs) with 95% Confidence Intervals (95% CIs). We pooled continuous outcomes using the inverse-variance method and reported Mean Differences (MDs) with 95% CIs. We set statistical significance at p < 0.05. Given the anticipated clinical and methodological heterogeneity across RCTs, we conducted all analyses using a random-effects model with restricted maximum likelihood estimation. We assessed heterogeneity using the Cochran *Q*-test and the I² statistic, classifying it as mild (I² < 25%), moderate (I² 25%–49%), or substantial (I² ≥ 50%).^25^

In accordance with previous evidence, the Minimal Clinically Important Difference (MCID) was set at 10 mm for pain scores and at 40 mg of OME in 24 hours for opioid consumption.^26^^,^^27^ All analyses were performed in the “meta” package of R 4.1.2 (R Foundation for Statistical Computing, Austria). Lastly, we performed TSA for every included outcome (version 0.9.5.10, Copenhagen Trial Unit, Denmark), with a type I error of 0.05 and a type II error of 0.20 (80% power), and applied the O'Brien-Fleming alpha-spending function for the *Z*-score.^28^

## Results

### Study selection and characteristics

The search identified 345 records. After removing 171 duplicates, 174 records underwent title and abstract screening; 150 were excluded. Full-text assessment was performed for 24 studies (Supplementary Table 3), and 7 were included in the meta-analysis (Fig. 1), comprising 591 adults, of whom 295 (49.9%) were randomized to receive methadone. The baseline characteristics of these RCTs are shown in Table 1. Given the variability in anesthetic regimens, including methadone dosing and timing, as well as endpoint definitions, we provided trial-level protocol details for each study (Supplementary Table 2). The reasons for full-text exclusions are itemized in Figure 1. None of the included trials and cited references underwent retraction, withdrawal, expression of concern, or correction in the source database or on the publisher's website, as of October, 2026.

**Figure 1:**
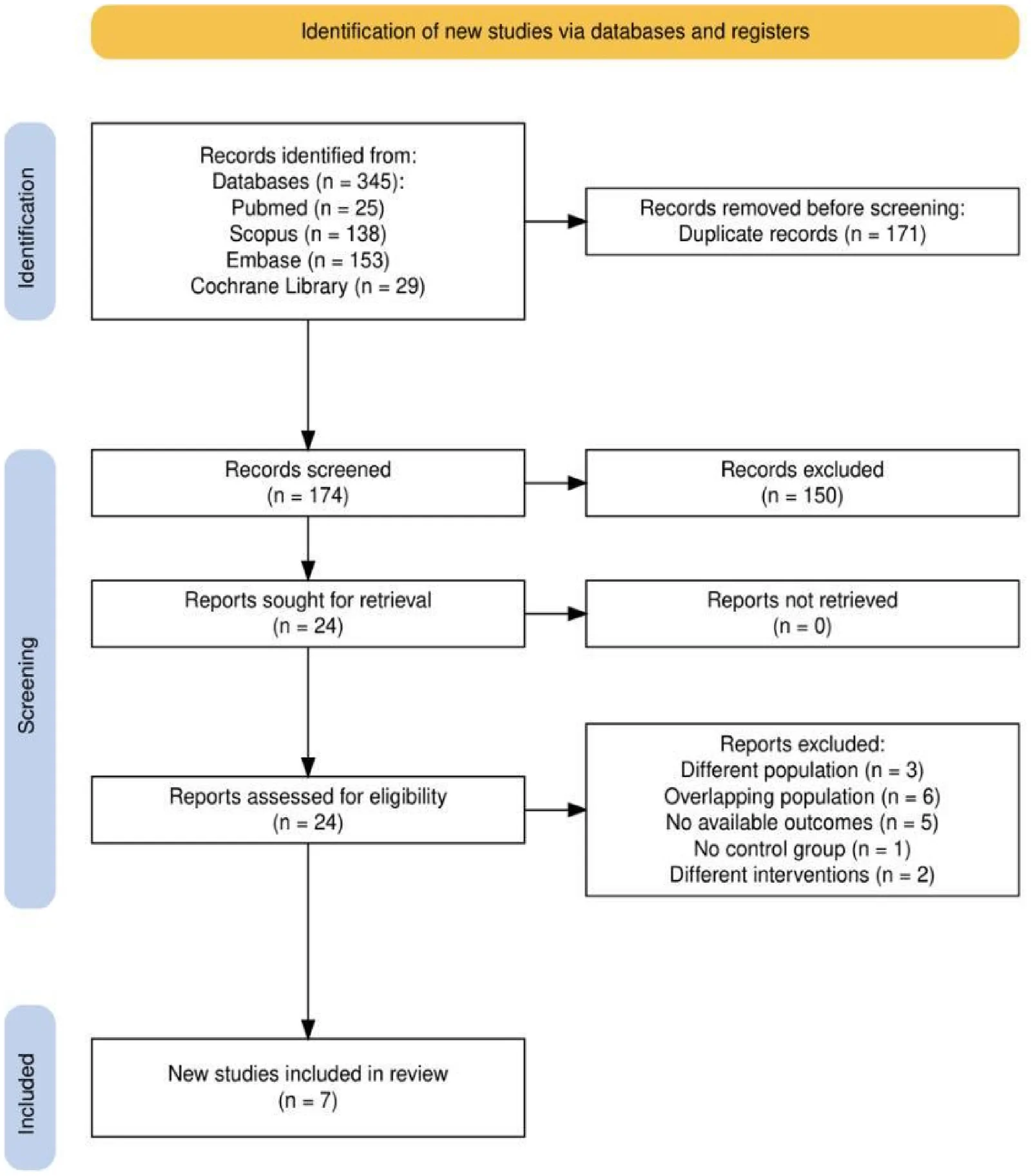
Preferred reporting items for systematic reviews and meta-analyses flow diagram of study selection.

**Table 1:** Qualitative characteristics of the included studies.

Author, year	Country	Type of Surgery	Patients, n Intervention Control	Male, n (%) Intervention Control	Age, years^a^ Intervention Control	BMI^a^ Intervention Control (kg.m^-2^)	Duration of surgery, min^a^ Intervention Control	Intervention Control
Arriaza, 2021^1^	Chile	Laparoscopic Cholecystectomy	39	15 (35.7%)	49.4 ± 14.2	28 ± 3.4	60.2 ± 19.4	Methadone 0.08 mg.kg^-1^
			39	14 (32.6%)	54.4 ± 12.8	27.7 ± 3.4	59.4 ± 19.9	Morphine 0.08 mg.kg^-1^
Simoni, 2009^2^	Brazil	Laparoscopic Cholecystectomy and Hiatal Hernia Repair	42	N/R	45 ± 12	79 ± 16	65 ± 20	Methadone 0.1 mg.kg^-1^
			42		40 ± 10	76 ± 17	57 ± 15	Placebo (Normal Saline)
Pontes, 2021^15^	Brazil	Laparoscopic Bariatric	69	15 (22%)	36.2 ± 10.5	40.0 ± 4.2	53 (48–60)	Methadone 0.1 mg.kg^-1^
			68	19 (28%)	34.4 ± 8	39.6 ± 3.7	53 (47–61)^b^	Morphine 0.1 mg.kg^-1^
Freire, 2025^16^	Brazil	Laparoscopic Bariatric	16	1 (6.2%)	59.01 ± 12.50	42.1 ± 7.1	264 ± 78	Methadone 10 mg diluted in 100 mL saline
			18	5 (17.8%)	57.78 ± 9.43	44.8 ± 5.3	258 ± 66	Placebo 100 mL Normal saline
Green, 2024^17^	Australia	Gynecological Laparoscopy	35	0	33.17 ± 10.49	25.60 ± 4.20	N/R	Methadone 10 mg at induction (or 0.15 mg.kg^-1^ if < 10 lt; 10 mg)
			34	0	32.74 ± 9.51	25.81 ± 4.09		Fentanyl, morphine, or oxycodone (mean intraoperative dose ≈ 47 mg OME).
Friesgaard, 2023^18^	Denmark	Laparoscopic Hysterectomy	64	0	47.4 (45.7–49.1)	26.9 (25.7–28.0)	108 (90–130)	Methadone 0.2 mg.kg^-1^
			63	0	46.6 (44.6–48.6)	27.1 (26.3–28.7)^a^	107 (91–120)^b^	Morphine 0.2 mg.kg^-1^
Moro, 2019^19^	Brazil	Laparoscopic Cholecystectomy	31	3 (9.7%)	48.0 ± 11.3	28 (26–33)	60 (60–75)	Methadone 0.1 mg.kg^-1^
			31	7 (22.6%)	42.3 ± 10.2	28 (24–32)^b^	60 (50–70)^b^	Morphine 0.1 mg.kg^-1^

N, Number; BMI, Body Mass Index; IQR: interquartile range; kg, kilograms; lt: liters; m, meters; min, minutes; mg, milligrams; mL, milliliters; N/R, Not Reported; OME, Oral Morphine Equivalents; SD: standard deviation.

a Mean ± SD.

b Median (IQR).

### Pooled analysis

Cumulative opioid consumption within 24 hours, measured in OME, was significantly lower in the methadone group (MD = -1.62; 95% CI -2.75 to -0.50; p = 0.004; I² = 55%; three trials; 239 patients, Fig. 2). The incidence of PACU opioid use was also reduced in the methadone group (RR = 0.69; 95% CI 0.53–0.90; p = 0.006; I² = 52.6%; four trials; 365 patients, Fig. 3A). The risk of postoperative hypoxia decreased in patients receiving methadone (RR = 0.24; 95% CI 0.06–0.91; p = 0.036; I² = 0%; three trials; 233 patients, Fig. 3B). Pain scores were notably lower in the methadone group at 30 minutes (MD = -1.21; 95% CI -1.91 to -0.51; p = 0.0007; I² = 47.7%; three trials; 274 patients, Fig. 3C) and at 60 minutes (MD = -0.41; 95% CI -0.80 to -0.03; p = 0.0361; I² = 0%; four trials; 336 patients, Fig. 3D).

**Figure 2:**
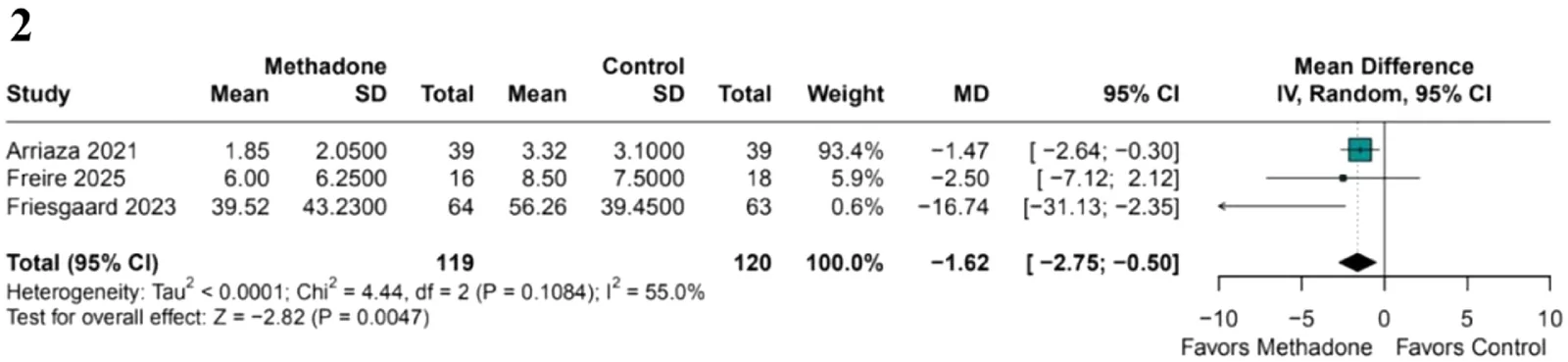
Forest plot for cumulative opioid use in the first 24 hours. MD, Mean Difference; CI, Confidence Interval.

**Figure 3:**
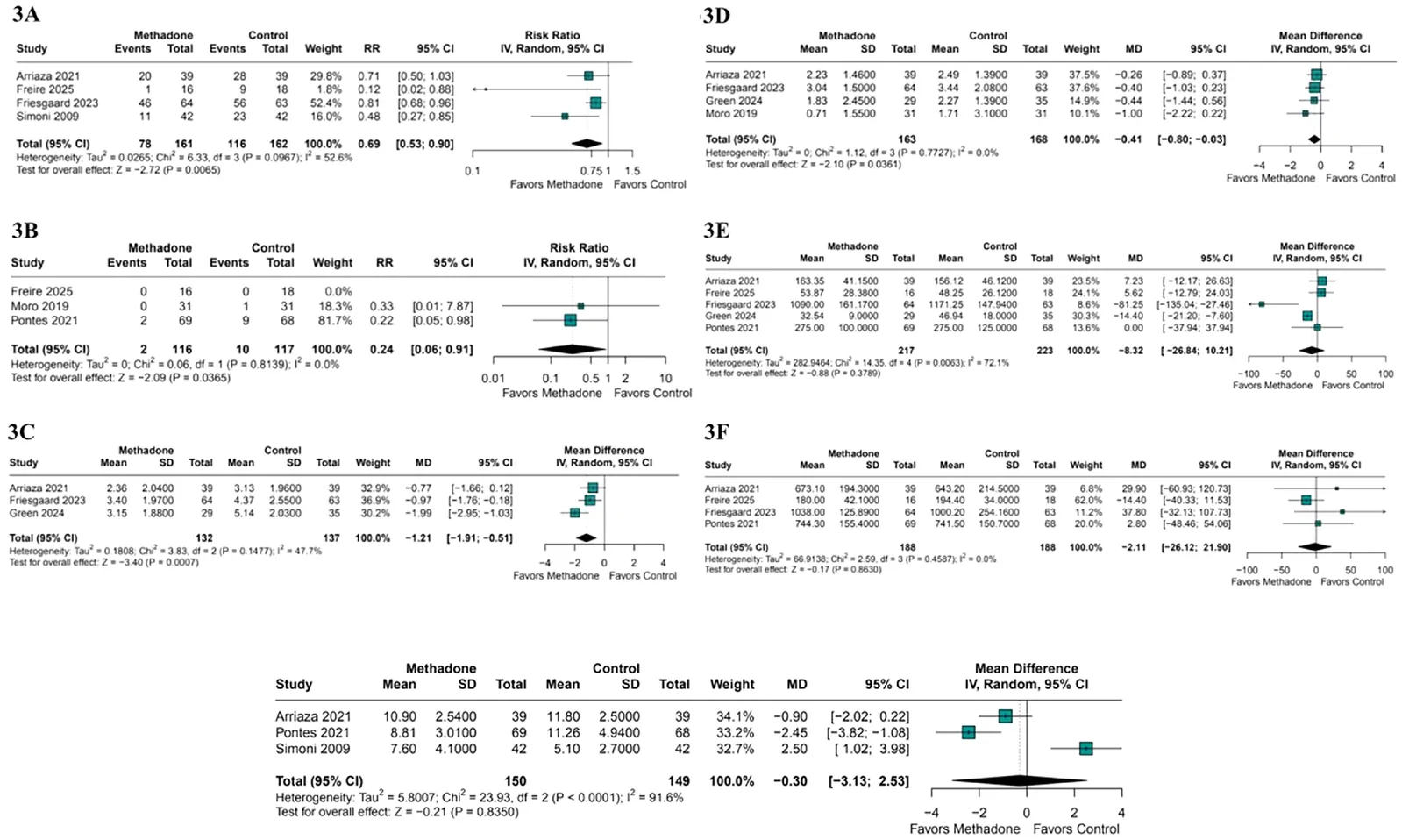
(A) Forest plot for the incidence of Post-Anesthesia Care Unit (PACU) opioid use. (B) Forest plot for the risk of postoperative hypoxia. (C) Forest plot for pain scores at 30 minutes. (D) Forest plot for pain scores at 60 minutes. (E) Forest plot for intraoperative opioid consumption. (F) Forest plot for intraoperative propofol consumption. RR, Risk Ratio; MD, Mean Difference; CI, Confidence Interval; SD: Standard Deviation.

No significant differences were observed between groups in intraoperative opioid consumption (MD = -8.32; 95% CI -26.84 to 10.21; p = 0.37; I² = 72.1%; five trials; 411 patients, Fig. 3E), intraoperative propofol consumption (MD = -2.11; 95% CI -26.12 to 21.9; p = 0.863; I² = 0%; four trials; 376 patients, Fig. 3F), emergence time (MD −0.30; 95% CI -3.13 to 2.53; p = 0.835; I² = 91.6%; three trials; 341 patients, Fig. 4A), PACU length of stay (MD = -11.22; 95% CI -25.69 to 3.25; p = 0.128; I² = 89.1%; three trials; 234 patients, Fig. 4B), and pain at PACU arrival (MD = -2.47; 95% CI -5.49 to 0.55; p = 0.109; I² = 97.2%; four trials; 243 patients, Fig. 4C). Pain scores at 24 hours (MD = -0.44; 95% CI -1.68 to 0.8; p = 0.486; I² = 86.6%; four trials; 301 patients, Fig. 4D) and patient satisfaction (MD = -0.00; 95% CI -0.24 to 0.24; p = 0.987; I² = 0.6%; three trials; 326 patients, Fig. 4E) were also not significantly different. Similarly, the incidence of postoperative nausea and vomiting (RR = 0.67; 95% CI 0.41–1.1; p = 0.114; I² = 0%; three trials; 238 patients, Fig. 5A) and respiratory depression (RR = 1.54; 95% CI 0.83–2.85; p = 0.173; I² not applicable; two trials, 69 patients, Fig. 5B) showed no significant differences between groups.

**Figure 4:**
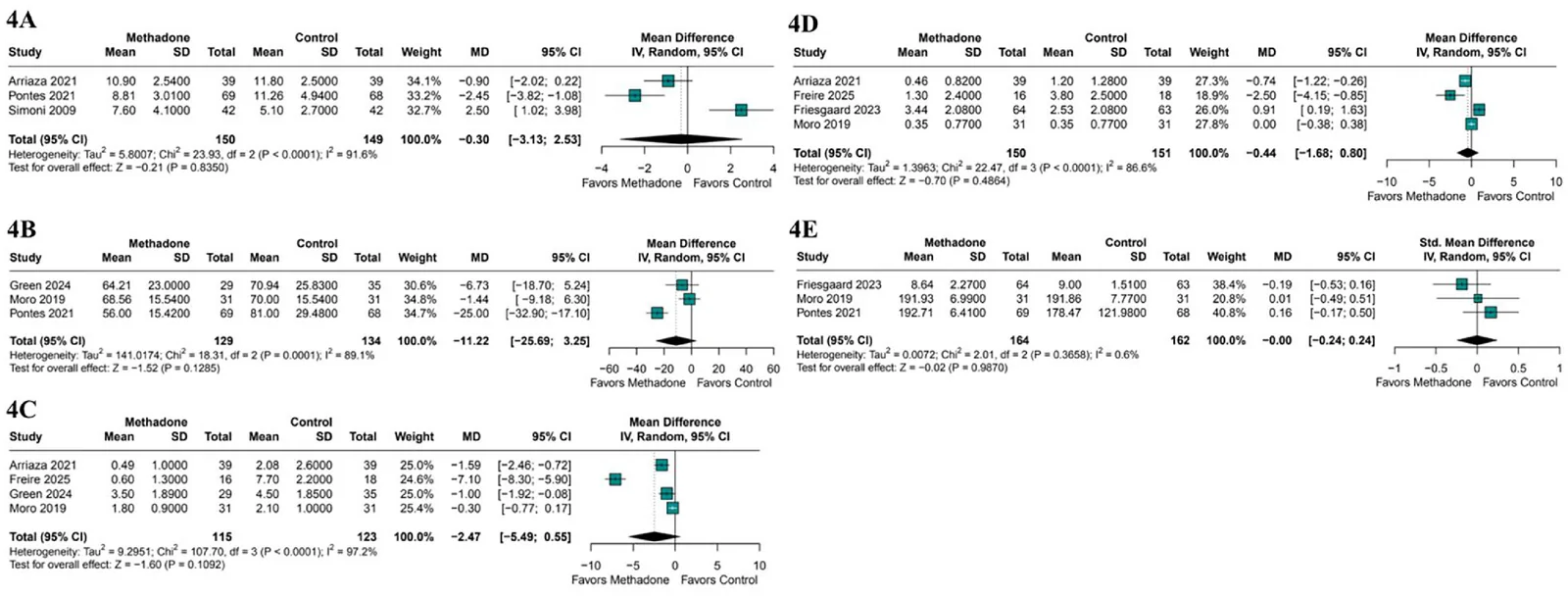
(A) Forest plot for emergence time, (B) Forest plot for PACU stay, (C) Forest plot for pain at PACU arrival, (D) Forest plot for pain scores at 24 hours, (E) Forest plot for patient satisfaction. MD, Mean Difference; CI, Confidence Interval; SD: Standard Deviation.

**Figure 5:**
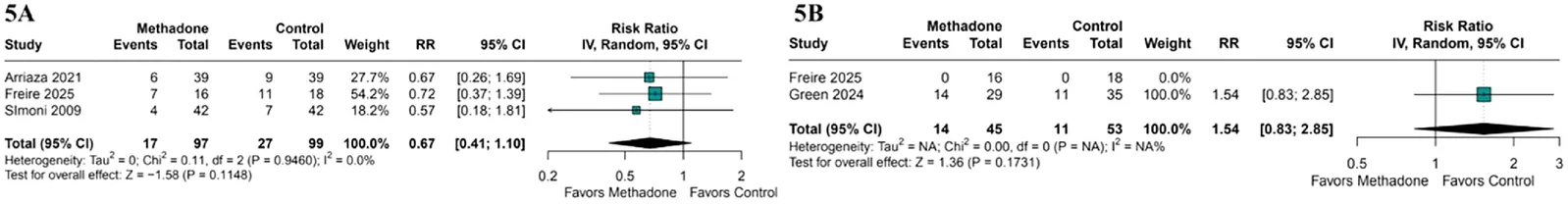
(A) Forest plot for the incidence of postoperative nausea and vomiting. (B) Forest plot for the incidence of respiratory depression. RR, Risk Ratio; CI, Confidence Interval.

### Sensitivity analyses

Leave-one-out sensitivity analyses revealed that the statistical significance for 24-hour opioid consumption was lost when Arriaza et al.^1^ or Freire et al.^16^ were excluded, while removing Friesgaard et al.^18^ reduced heterogeneity to 0% and maintained statistical significance. For the emergence time, excluding Simoni et al.^2^ resulted in a statistically significant favoring of methadone. Additionally, removing Pontes et al.^15^ rendered hypoxia non-significant. For PACU opioid use, excluding Arriaza et al.,^1^ caused a loss of significance. Intraoperative opioid and propofol consumption remained unaffected by omitting any individual study. Excluding Freire et al.^16^ made pain at PACU arrival statistically significant (MD = -0.89; 95% CI -1.67 to -0.11). Pain at 30 and 60 minutes and at 24 hours, PACU length of stay, PONV, and patient satisfaction remained consistently significant and in the same direction across all sensitivity analyses. Detailed leave-one-out results are available in the Supplementary Figure 1.

### Trial sequential analysis

As shown in Figure S2A, the Trial Sequential Analysis (TSA) indicated that the cumulative *Z*-curve for 24-hour opioid consumption crossed the conventional significance boundary favoring methadone, but did not reach the monitoring boundary or the required information size. Similarly, for hypoxia, as shown in Figure S2B, the *Z*-curve crossed the conventional boundary in favor of methadone but did not meet either the monitoring boundary or the required information size. Regarding pain outcomes, the *Z*-curve at 30 minutes crossed the required information size (Fig. S2C). In contrast, at 60 minutes, it crossed only the conventional boundary favoring methadone, without reaching the monitoring boundary or the required information size (Fig. S2D). Conversely, for the incidence of patients requiring postoperative opioids, the *Z*-curve crossed the conventional boundary favoring methadone and reached the required information size (Fig. S2E).

### Risk of bias

Five of our included studies were deemed to have a low risk of bias.^1^^,^^15^^,^^16^^,^^18^^,^^19^ Two studies had some concerns: Green et al.^17^ due to deviations from the intended intervention, and Simoni et al.^2^ because of potential bias in the reported results (Fig. S3).

We did not evaluate publication bias because none of the pooled outcomes included at least ten studies. Funnel plots and statistical tests for publication bias are unreliable when few studies are available, and the Cochrane Handbook recommends using these methods only when at least 10 studies are included to ensure adequate power.^29^^,^^30^

### Certainty of evidence

The risk-of-bias assessment indicates that the quality of the included RCTs was reasonable. However, the GRADE assessment also demonstrated that the overall certainty of the evidence in this meta-analysis was moderate to low, mainly due to heterogeneity and imprecision (Supplementary Table 4).

## Discussion

In this systematic review, meta-analysis, and TSA including 591 patients from 7 RCTs, we found that methadone was associated with decreased 1) Cumulative opioid consumption in the first 24 hours, 2) Incidence of hypoxia, 3) Incidence of opioid use in PACU, and 4) Pain scores at PACU at 30-min and 60-min, although some of these results did not meet clinical benefit and showed low certainty of evidence. We observed no differences between groups regarding emergence time, incidence of PONV, incidence of respiratory depression, intraoperative opioid use, intraoperative propofol use, PACU length of stay, patient satisfaction, and pain scores on PACU arrival and at 24h.

Our analysis showed that methadone significantly decreased the total opioid use 24 hours after laparoscopic surgery. However, the reduction was much smaller than the MCID suggested by Versyck (30 mg OME) and Laigaard (10 mg IV morphine equivalents). While MCID thresholds vary by context and no universal standard exists, the small effect observed here likely does not provide a meaningful clinical benefit.^27^^,^^31^ The TSA confirmed that the result was statistically significant but did not cross the monitoring boundaries or achieve the required information size, indicating a potential risk of a type I error.^32^

Sensitivity analysis for cumulative opioid use in 24 hours revealed that removing Friesgaard's^18^ study reduced heterogeneity to 0% while maintaining statistical significance. This reduction likely reflects methodological and clinical differences between this study and the other two trials. Friesgaard et al.^18^ focused on laparoscopic hysterectomy, used an equal-potency comparator (morphine vs. methadone), and administered the study drug 60 minutes before extubation, unlike the other RCTs that administered it at induction. Additionally, opioid use was recorded as total 24-hour OME from both PACU and ward, following a standardized rescue protocol. In contrast, Arriaza et al.^1^ only measured PACU morphine use and reported it as 24-hour opioid consumption, since institutional policy restricted opioid use outside PACU, and ward regimens, including nalbuphine, influenced Freire et al.’s^16^ 24-hour totals. Removing Friesgaard et al.^18^ aligns the trials regarding design and outcome measurement, thus removing these sources of heterogeneity. This result aligns with Machado et al., who reported a mean difference of -8.42 mg IV morphine equivalents when comparing methadone with other opioids across surgical populations.^8^ Although this effect was also below MCID thresholds, it was larger than ours, likely reflecting the inclusion of more painful procedures such as spine surgery, which have higher postoperative opioid requirements.^33^ In contrast, laparoscopic procedures like those in our review are less invasive and generally involve lower baseline pain and opioid use, limiting the measurable opioid-sparing potential of methadone.^34^^,^^35^ Nonetheless, we acknowledge that some key effects lose statistical significance when individual studies are removed, which might indicate fragility of the results, especially given that our results were below the MCID and that the number of studies was insufficient to conduct further subgroup analyses by procedure type.

Our analysis also showed that intraoperative methadone significantly reduced the risk of postoperative hypoxia, with no heterogeneity across studies (76% relative risk reduction). The TSA confirmed that this finding reached conventional statistical significance but did not cross the monitoring boundaries or meet the required information size, also indicating a potential risk of type I error and thus reducing the certainty of the evidence, likely due to the low pooled number of events across included studies. A plausible explanation for the possible reduction in hypoxia might be the lower cumulative opioid consumption observed in the methadone group, since opioids are known to cause respiratory complications. This interpretation is supported by RCTs where morphine recipients exhibited higher postoperative sedation and required more supplemental opioids compared to those given methadone.^15^^,^^19^ Although a cohort study by Carlé et al. found no difference in respiratory depression between methadone and morphine, our pooled findings contradict that, despite a limited number of included studies.^36^ Given that methadone and morphine have similar potency according to equianalgesic estimates, the most likely explanation is that reduced opioid consumption in the methadone group might indirectly translate into fewer hypoxic episodes.^37^^,^^38^

Furthermore, while the incidence of respiratory depression did not differ between groups (despite including only 2 RCTs), the higher frequency of hypoxia in the morphine group may indicate that episodes of respiratory depression were more severe in these patients, even if they were not more common. Overall, these findings suggest that intraoperative methadone may provide a respiratory safety benefit through opioid-sparing effects. However, the certainty of this evidence remains limited due to the TSA results, the low number of included trials and pooled events, and the broad definitions of hypoxia and respiratory depression across the included trials (Supplementary Table 2). We understand that methadone dosing and timing varied widely (i.e., four studies at induction and two studies before extubation; fixed *vs*. weight-based dosing; Supplementary Table 2), which may have influenced early analgesic or respiratory outcomes.

We observed no statistically significant differences in pain scores at PACU arrival. In contrast, pain was significantly reduced in the methadone group at 30 and 60 minutes, probably due to the time to methadone's peak analgesic effect (nearly 30 minutes after intravenous infusion).^6^ The MCID for postoperative pain is 10-mm on a 100-mm VAS (equivalent to 1-point on a 0–10 NRS), and the Patient Acceptable Symptom State (PASS) is 33 mm.^26^ Across all included trials, mean pain scores at PACU arrival, 30-min, 60-min, and 24 hours were consistently well below the PASS threshold, typically ranging between 0.5 and 3 on the NRS. Therefore, while the reduction at 30 minutes exceeded the MCID and was both statistically and clinically significant, the difference at 60 minutes, although statistically significant, was below the MCID threshold and unlikely to be clinically relevant. This finding highlights the importance of differentiating statistically significant from clinically significant results using MCID, a growing trend in anesthesiology research that aids the interpretability and applicability of findings.^26^

The TSA confirmed that the 30-minute effect was statistically robust and exceeded the required information size, reducing the risk of a type I error. In contrast, the TSA for 60 minutes confirmed statistical significance but did not cross monitoring boundaries or reach the information size, leaving uncertainty about the effect's reliability. The absence of significance at PACU arrival may be due to residual effects of intraoperative opioids or early multimodal analgesia obscuring differences between groups.^39^, ^40^, ^41^ Similarly, the lack of significance at 24 hours probably results from the short, minimally painful nature of laparoscopic procedures, routine use of non-opioid multimodal regimens, and the possibility that methadone’s long half-life offered no additional benefit once background pain scores had already dropped below clinically significant levels in both groups.^35^

Finally, although previous reviews suggest that the inclusion of methadone within Enhanced Recovery After Surgery (ERAS) protocols appears to reduce opioid consumption and lead to a significantly increased analgesic effect,^42^ some evidence suggests that the use of multimodal anesthesia may also dilute or increase methadone’s apparent effect.^43^ A previous meta-analysis of 929 patients assessing intraoperative methadone found that its analgesic benefits may extend up to 72 hours postoperatively;^8^ however, this study included an even higher variability of procedures and patients (i.e., pediatric and cardiac surgeries, ambulatory procedures), and the authors did not assess the MCID for most endpoints. In our study, we limited procedures to laparoscopic surgeries to reduce expected heterogeneity in baseline and postoperative pain burdens; however, variability in anesthetic regimens, methadone timing, and institutional PACU protocols may have contributed to variability in the results. In addition, although most studies included gynecological and biliary surgeries, we could not perform subgroup analyses because there were insufficient studies to pool by procedure. Nonetheless, despite this dispersion, the MCID findings suggest that methadone's role in pain management seems to be limited to the early postoperative periods, and further studies are warranted to better address its impacts on adverse events, such as QTc prolongation or, most importantly, respiratory outcomes (i.e., delayed respiratory depression), given the limited number of reporting trials.

Nonetheless, this study has some limitations. First, we included a small sample size of 591 patients across seven RCTs, and no single outcome was reported in all trials; the small number of studies per outcome also prevented subgroup analyses and meta-regression to examine the effect of methadone dosing. Furthermore, the GRADE assessment was mostly low to moderate, with several outcomes showing limited reporting of adverse events and heterogeneity due to differences in surgical procedures, populations, and methodologies; we further increased variability by including different laparoscopic surgeries, which might restrict generalizability. In addition, although we employed the “backward snowballing” technique to identify additional studies, we did not search the gray literature, which might have contributed to publication bias. Lastly, TSA indicated that most outcomes did not reach the required information size, suggesting a risk of type I error and highlighting the need for larger studies with more participants.

## Conclusion

Intraoperative methadone in laparoscopic surgery was associated with reduced early postoperative pain scores, reduced opioid use, and less hypoxia without evidence of increased adverse events. However, most effects were small, the decrease in opioid consumption did not reach the minimal clinically important difference, and the certainty of evidence was limited by heterogeneity, imprecision, and TSA findings. Future well-powered trials with standardized methadone dosing and outcome definitions are needed to determine if these early benefits translate into consistent clinical improvements.

## Data availability statement

The extracted study-level dataset, opioid conversion table, GRADE evidence profile, Trial Sequential Analysis settings, and statistical analysis code used to reproduce the analyses are provided in the Supplementary Material.

## Declaration of artificial intelligence (AI) use

The authors did not use AI or AI-assisted technologies in writing this manuscript.

## Authors’ contributions

All authors contributed to the study's conception and design. Rafael A. Lombardi, Julio S. Curi, Carlos Martins, and Matheus Arraes performed material preparation, data collection, and analysis. Rafael A. Lombardi and Julio S. Curi wrote the first draft of the manuscript. All authors critically reviewed previous versions of the manuscript. All authors read and approved the final manuscript. All authors agree to be accountable for all aspects of the work.

## Funding

The authors did not receive support from any organization for the submitted work.

## Conflicts of interest

The authors declare no conflicts of interest.

## Appendix Supplementary materials

Image, application 1

## Footnotes

Supplementary material associated with this article can be found in the online version at doi:10.1016/j.bjane.2026.844848.
