# Supplementary material for: Intraoperative methadone in adult laparoscopic surgery: a systematic review and meta-analysis of randomized trials
Source: Braz J Anesthesiol. 2026 Oct 15;76(6):844848. doi: 10.1016/j.bjane.2026.844848 (PMC13760108; doi:10.1016/j.bjane.2026.844848)
Supplement: Supplementary file 1 [file mmc1.docx]

**BJAN-D-25-00817_Supplementary Material**

**Supplementary Material For:**

Intraoperative methadone in adult laparoscopic surgery: a systematic review and meta-analysis of randomized trials

**Supplementary File 1** Figures and Tables.

**Supplementary Figure 1**.

**1A.** Leave-one-out analysis for the outcome of 24-hour opioid consumption.

**1B.** Leave-one-out analysis for the outcome time to emergence.

**1C**. Leave-one-out analysis for the outcome of hypoxia.

**1D.** Leave-one-out analysis for the outcome of incidence of post-anesthesia care unit (PACU) opioid use.

**1E.** Leave-one-out analysis for the outcome of intraoperative opioid use.

**1F.** Leave-one-out analysis for the outcome of intraoperative propofol use.

**1G**. Leave-one-out analysis for the outcome of pain at PACU arrival.

**1H.** Leave-one-out analysis for the outcome of pain scores at 30 minutes.

**1I.** Leave-one-out analysis for the outcome of pain at 60 minutes.

**1J.** Leave-one-out analysis for the outcome of pain at 24 hours.

**1K**. Leave-one-out analysis for the outcome of PACU stay.

**1L.** Leave-one-out analysis for the outcome of postoperative nausea and vomiting.

**1M.** Leave-one-out analysis for the outcome of patient satisfaction at 24 hours.

**Supplementary Material** **Figure 2** Trial sequential analyses.

**2A**. Trial sequential analysis for cumulative opioid consumption at 24 h.

**2B.** Trial sequential analysis for hypoxia.

**2C.** Trial sequential analysis for static pain score at 30 minutes.

**2D**. Trial sequential analysis for static pain score at 60 minutes.

**2E.** Trial sequential analysis for patients requiring postoperative opioids.

**Supplementary Figure 3** Cochrane Risk of Bias 2 (RoB 2) tool assessment.

**Supplementary Table 1** Search strategy for each database.

| **Database** | **Search Strategy** |
| --- | --- |
| PubMed | ("laparoscopie"[All Fields] OR "laparoscopy"[MeSH Terms] OR "laparoscopy"[All Fields] OR "laparoscopies"[All Fields] OR ("laparoscopes"[MeSH Terms] OR "laparoscopes"[All Fields] OR "laparoscope"[All Fields] OR "laparoscopical"[All Fields] OR "laparoscopically"[All Fields] OR "laparoscopics"[All Fields] OR "laparoscopy"[MeSH Terms] OR "laparoscopy"[All Fields] OR "laparoscopic"[All Fields])) AND ("methadon"[All Fields] OR "methadone"[Supplementary Concept] OR "methadone"[All Fields] OR "methadone"[MeSH Terms] OR "methadone s"[All Fields]) |
| Embase | ('laparoscopy'/exp OR laparoscopy OR laparoscopic) AND ('methadone'/exp OR methadone) |
| Scopus | (laparoscopy OR laparoscopic) AND methadone |
| Cochrane | (laparoscopy OR laparoscopic) AND methadone |

**Supplementary Table 2** Anesthesia technique and outcome definitions within each included study.

| **Study** | **Methadone Regimen** | | **Comparator Regimen** | **Anesthesia Technique** | **Co-analgesics (Intraop/Postop)** | **PACU Rescue Protocol** | **Hypoxia** |  | **Respiratory depression** | **PONV** |
| --- | --- | --- | --- | --- | --- | --- | --- | --- | --- | --- |
| Green 2024 | Single intraoperative dose of 10 mg IV at induction (or lower doses were allowed at the anesthetist’s discretion (e.g., in smaller patients) at 0.15 mg/kg, provided this was less than 10 mg  (In the methadone group, the only opioid allowed was methadone) | Short-acting opioids (fentanyl, morphine, or oxycodone) are chosen at the anesthetist’s discretion. | | Premedication, anesthesia induction, and maintenance were at the attending anesthetist’s discretion. | Paracetamol, Parecoxib, Ketamine, Pregabalin 150 mg preoperatively | PACU: Pain protocols for fentanyl, morphine, and oxycodone were available for use. | NA |  | Respiratory rate of less than 10 breaths per minute for 10 min | NA |
| Arriaza 2021 | 0.08 mg/kg (calculated based on lean body mass) IV at start of surgery | Morphine 0.08 mg/kg (calculated based on lean body mass) IV at the start of surgery | | Induction and maintenance anesthesia were based on remifentanil and propofol, titrated to attain a bispectral index value between 40 and 60. | Following Induction: Dexamethasone 8 mg + Ketoprofen 100 mg + Metamizole 2 g. | Post-operative analgesia using a continuous infusion of Ketoprofen 100 mg + Metamizole 3 mg  PACU: If NRS ≥ 4: morphine 1 mg  In the ward: Ketorolac 30 mg | NA |  | NA | NR |
| Freire 2025 | Methadone 10 mg IV diluted in 100 mL of saline (≈0.1–0.2 mg/kg IBW +20%) after induction | 100 mL of 0.9% saline | | Anesthesia was induced with propofol, sufentanil, and a neuromuscular blocker, either cisatracurium or rocuronium. Titrated NMB doses were administered to ensure optimal surgical relaxation  Anesthesia maintenance: sevoflurane at 1.8-2%  If hypotension (reduction of 20% or more from the initial blood pressure): boluses of either metaraminol (0.5 mg) or ephedrine (5 mg), based on clinical indications. | Beginning of procedure: dexamethasone 4 mg  Conclusion of procedure: Metamizole 2 g + ondasetron 8 mg + Tenoxican 40 mg)  No other medications that interfere with pain control, such as remifentanil, esketamine, dexmedetomidine, magnesium sulfate, and lidocaine. | IV morphine 1 mg every 1 mg, every five minutes  In the ward, the patients received a fixed dose of metamizole 1 g IV every six hours, tenoxicam 40 mg once a day, and nalbuphine 10 mg IV up to every six hours was prescribed for pain relief, as needed. | Sustained desaturation or hypoventilation with less than 90% oxygen |  | NR | NR |
| Friesgaard 2023 | 0.2 mg/kg IV - 60 min before extubation | Morphine 0.2 mg/kg IV - 60 minutes before extubation | | Anesthesia was induced and maintained using a standard protocol with propofol and remifentanil (doses adjusted according to bispectral index values and clinical judgement of anesthesia depth). Tracheal extubation took place in the operating theatre before the patient was transferred to the PACU. | On the morning of surgery: acetaminophen 1000 mg + ibuprofen 400 mg. Following anesthetic induction: dexamethasone 8 mg IV. In some cases, infiltration of 2.5% bupivacaine (5 mL) into the skin and fascia before incision, and intraperitoneal injection of 2% ropivacaine (50 mL) before closure, were also standard. | Moderate pain - NRS > 3: morphine IV (initial dose 0.1 mg kg^-1^ [< 75 yr] and 0.05 mg kg^-1^ [> 75 yr]) and titrated (50% of initial dose) every 10 min until NRS 3. Supplementary oral morphine was administered (10 mg [< 75 yr] or 5 mg [> 75 yr]) when necessary. If there was an inadequate response to morphine, IV oxycodone and oral oxycodone were administered following the same procedure and dose as morphine. In case of severe pain intensity (NRS > 6), morphine was supplemented with IV fentanyl (single dose of IV fentanyl 50 mg [adults between 18 and 75 yr] or IV fentanyl 25 mg [adults < 75 yr, weight < 50 kg, or kidney disease]). Opioid analgesia was supplemented with oral paracetamol and ibuprofen. Dexamethasone, ondansetron, droperidol, and cyclizine were used for prophylaxis and treatment of postoperative nausea and vomiting (PONV). | NA |  | NA | NA |
| Moro 2019 | Methadone 0.1 mg/kg IV after induction | Morphine 0.1 mg/kg IV after induction | | Induction: 1% lidocaine 30 mg IV, remifentanil 0.5 μg kg over 3 min, propofol 2.0 mg kg, rocuronium 0.6 mg kg +  Maintenance: CI remifentanil 0.3 μg kg + CI propofol 4 to 6 mg kg | Infiltration: 0.25% bupivacaine 5 mL  After induction: Dexamethasone 8 mg + ketoprofen 100 mg  Before the end of surgery: ondansetron 4 mg + metamizole 1g | Every 6 h: ketoprofen 100 mg IV + metamizole 30 mg kg^−1^ (maximum 1 g)  If pain score > 3: Tramadol 100 mg IV | NR |  | NA | NA |
| Pontes 2021 | 0.1 mg/kg (based on IBW) IV 20 min before the end of surgery | Morphine 0.1 mg/kg (based on IBW) IV 20 min before the end of surgery | | No premedication  Induction: propofol 2-3 mg.kg(IBW)^-1^; remifentanil infused continuously at 0.1 μg.kg^-1^(IBW).min + rocuronium 1.2 mg.kg(IBW)^-1^ + lidocaine 1.5 mg.kg(IBW)  Intraoperative: Propofol TCI (Marsh) propofol 1–4 μg.ml + continuous infusion of remifentanil 0.1–0.2 μ[g.kg](http://g.kg) IBW.min), targeting mean blood pressure within 20% of baseline values and a bispectral index within the range of 40–60.  At the end of the procedure, 5 mL of levobupivacaine 0.5% was infiltrated into each of the skin incision sites. | Immediately after induction: Dexamethasone 10 mg, Omeprazole 40 mg, Parecoxib 40 mg  Twenty minutes before the end of the procedure: ondansetron 8 mg, metamizole 3 g | If NRS ≥ 4: morphine 2 mg IV  If the RR ≤ 8: Naloxone 40 mcg IV  (During the postoperative period on the ward, all patients were given: omeprazole 40 mg every 8 h; ondansetron 4 mg every 8 h; and metoclopramide 10 mg every 8 h. For analgesia, they received metamizole 1 g every 6 h and parecoxib 40 mg every 24 h. If refractory pain was present, morphine 2 mg was administered intravenously every 15 min until NRS was < 4.) | SpO_2_ ≤ 90% |  | Respiratory rate ≤ 8 | NA |
| Simoni 2009 | 0.1 mg/kg IV before the start of surgery | Normal saline | | Induction: Remifentanil TCI 6.0 μg.mL + Propofol TCI 4.0 μg.mL + BIS + cisatracurium. After intubation: Remifentanil TCI 3.0 μg.mL + Propofol TCI 2.0 to 3.5 μg.mL  Intraoperative: Remifentanil TCI 5.0 μg.mL | After puncture: Ketoprofen 1,5 mg/kg + Metamizole 30 mg/kg | If VAS ≥ 3, Intravenous tramadol (1.5 mg.kg^-1^) administered | NA |  | NA | NR |

Intraop: Intraoperative; Postop: Postoperative; PONV: Postoperative nausea and vomiting; IV: Intravenous; PACU: Post-Anesthesia Care Unit; TIVA: Total Intravenous Anesthesia; mg: Milligram; kg: Kilogram; NRS: Numeric Rating Scale; BP: Blood Pressure; VAS: Visual Analog Scale; NR: Not reported (the study assessed the outcome but did not report its definition); NA: Not available (the study did not assess the outcome); TCI: Target-controlled infusion; IBW: Ideal body weight; BIS: Bispectral Index.

**Supplementary Table 3** List of excluded studies, with reasons.

| **Study** | **Journal** | **Reason** |
| --- | --- | --- |
| Azari 2013 | Obstetrical and Gynecological Survey | Different population |
| Bastian 2019 | Minerva Anestesiologica | Different population |
| Kau 2023 | Diseases of the Colon and Rectum | Different population |
| EUCTR2018-004351-20-DK | Protocol | Overlapping population |
| ACTRN12620001131943 | Protocol | Overlapping population |
| NCT01833715 | Protocol | Overlapping population |
| NCT03045133 | Protocol | Overlapping population |
| NCT03908060 | Protocol | Overlapping population |
| RBR-54g48y | Protocol | Overlapping population |
| IRCT20190127042511N1 | Protocol | No available outcomes |
| NCT06536517 | Protocol | No available outcomes |
| Friesgaard 2019 | Acta Anaesthesiologica Scandinavica | No available outcomes |
| RBR-6j4kw7q | Protocol | No available outcomes |
| RBR-7hmxnkm | Protocol | No available outcomes |
| Pontes 2021 | Anaesthesia | No control group |
| Silva 2022 | Brazilian Journal of Anesthesiology | Wrong intervention |
| Khalili 2017 | Journal of Isfahan Medical School | Wrong intervention |

**Supplementary Table 4** GRADEpro Guideline Development Tool assessment.

| **Outcomes** | **№ of participants**  **(studies)** | **Risk of bias** | **Inconsistency** | **Indirectness** | **Imprecision** | **Certainty of the**  **evidence** **(GRADE)** |
| --- | --- | --- | --- | --- | --- | --- |
|  |  |  |  |  |  |  |
| Cumulative opioid use in the first 24 hours | 239 (3 studies) | No | Serious^a^ | No | Serious^b^ | ⨁⨁◯◯  Low^a,b^ |
| Time to emergence | 299 (3 studies) | No | Serious^a^ | No | Serious^b^ | ⨁⨁◯◯ Low^a,b^ |
| Hypoxia | 233 (3 studies) | No | Serious^a^ | No | Serious^b^ | ⨁⨁◯◯ Low^a,b^ |
| Incidence of post-anesthesia care unit (PACU) opioid use | 323 (4 studies) | No | Serious^a^ | No | Serious^b^ | ⨁⨁◯◯ Low^a,b^ |
| Incidence of post-operative nausea and vomiting | 196 (3 studies) | No | Serious^a^ | No | Serious^b^ | ⨁⨁◯◯ Low^a,b^ |
| Incidence of respiratory depression | 103 (2 studies) | No | No | No | Very serious^c^ | ⨁⨁◯◯ Low^c^ |
| Intraoperative opioid consumption | 445 (5 studies) | No | Serious^a^ | No | Very serious^c^ | ⨁◯◯◯ Low^a,c^ |
| Intraoperative propofol use | 376 (4 studies) | No | No | No | Serious^b^ | ⨁⨁⨁◯ Moderate^a^ |
| PACU stay | 268 (3 studies) | No | Serious^a^ | No | Serious^b^ | ⨁⨁◯◯ Low^a,b^ |
| Pain scores at 24 hours | 301 (4 studies) | No | No | No | Very serious^c^ | ⨁⨁◯◯ Low^c^ |
| Pain scores at 30 min | 274 (3 studies) | No | Serious^a^ | No | Serious^b^ | ⨁⨁◯◯ Low^a,b^ |
| Pain scores at 60 min | 336 (4 studies) | No | Serious^a^ | No | Very serious^c^ | ⨁◯◯◯ Low^a,c^ |
| Pain at PACU arrival | 243 (4 studies) | No | Serious^a^ | No | No | ⨁⨁⨁◯ Moderate^a^ |
| Patient satisfaction | 326 (3 studies) | No | Serious^a^ | No | Serious^b^ | ⨁⨁◯◯ Low^a,b^ |

PACU: Post-anaesthesia care unit. High certainty: We are very confident that the true effect lies close to that of the estimate of the effect. Moderate certainty: We are moderately confident in the effect estimate; the true effect is likely to be close to the estimate of the effect, but there is a possibility that it is substantially different. Low certainty: Our confidence in the effect estimate is limited; the true effect may be substantially different from the estimate of the effect. Very low certainty: We have very little confidence in the effect estimate; the true effect is likely to be substantially different from the estimate of effect.

^a^ The outcome exhibited substantial heterogeneity. Downgraded by one level due to inconsistency.

^b^ The outcome exhibited wide confidence intervals. Downgraded by one level due to imprecision.

^c^ The outcome exhibited very wide confidence intervals. Downgraded by one level due to imprecision.

**Supplementary Table 5** Extracted Study-Level Dataset.

**A.** Cumulative opioid consumption within 24 hours.

| study | author | mean_control | sd_control | n_control | mean_inter | sd_inter | n_inter |
| --- | --- | --- | --- | --- | --- | --- | --- |
| Arriaza 2021 | Arriaza | 3.32 | 3.1 | 39 | 1.85 | 2.05 | 39 |
| Freire 2025 | Freire | 8.5 | 7.5 | 18 | 6 | 6.25 | 16 |
| Friesgaard 2023 | Friesgaard | 56.26 | 39.45 | 63 | 39.52 | 43.23 | 64 |

**B.** Incidence of PACU opioid use.

| study | author | events_control | n_control | events_inter | n_inter |
| --- | --- | --- | --- | --- | --- |
| Arriaza 2021 | Arriaza | 28 | 39 | 20 | 39 |
| Freire 2025 | Freire | 9 | 18 | 1 | 16 |
| Friesgaard 2023 | Friesgaard | 56 | 63 | 46 | 64 |
| Simoni 2009 | Simoni | 23 | 42 | 11 | 42 |

**C.** Postoperative Hypoxia.

| study | author | events_control | n_control | events_inter | n_inter |
| --- | --- | --- | --- | --- | --- |
| Freire 2025 | Freire | 0 | 18 | 0 | 16 |
| Moro 2019 | Moro | 1 | 31 | 0 | 31 |
| Pontes 2021 | Pontes | 9 | 68 | 2 | 69 |

**D.** Pain score at 30 minutes.

| study | author | mean_control | sd_control | n_control | mean_inter | sd_inter | n_inter |
| --- | --- | --- | --- | --- | --- | --- | --- |
| Arriaza 2021 | Arriaza | 3.13 | 1.96 | 39 | 2.36 | 2.04 | 39 |
| Friesgaard 2023 | Friesgaard | 4.37 | 2.55 | 63 | 3.4 | 1.97 | 64 |
| Green 2024 | Green | 5.14 | 2.03 | 35 | 3.15 | 1.88 | 29 |

**E.** Pain score at 60 minutes.

| study | author | mean_control | sd_control | n_control | mean_inter | sd_inter | n_inter |
| --- | --- | --- | --- | --- | --- | --- | --- |
| Arriaza 2021 | Arriaza | 2.49 | 1.39 | 39 | 2.23 | 1.46 | 39 |
| Friesgaard 2023 | Friesgaard | 3.44 | 2.08 | 63 | 3.04 | 1.5 | 64 |
| Green 2024 | Green | 2.27 | 1.39 | 35 | 1.83 | 2.45 | 29 |
| Moro 2019 | Moro | 1.71 | 3.1 | 31 | 0.71 | 1.55 | 31 |

**F.** Intraoperative Opioid consumption.

| study | author | mean_control | sd_control | n_control | mean_inter | sd_inter | n_inter |
| --- | --- | --- | --- | --- | --- | --- | --- |
| Arriaza 2021 | Arriaza | 156.12 | 46.12 | 39 | 163.35 | 41.15 | 39 |
| Freire 2025 | Freire | 48.25 | 26.12 | 18 | 53.87 | 28.38 | 16 |
| Friesgaard 2023 | Friesgaard | 1171.25 | 147.94 | 63 | 1090 | 161.17 | 64 |
| Green 2024 | Green | 46.94 | 18 | 35 | 32.54 | 9 | 29 |
| Pontes 2021 | Pontes | 275 | 125 | 68 | 275 | 100 | 69 |

**G.** Intraoperative Propofol consumption.

| study | author | mean_control | sd_control | n_control | mean_inter | sd_inter | n_inter |
| --- | --- | --- | --- | --- | --- | --- | --- |
| Arriaza 2021 | Arriaza | 643.2 | 214.5 | 39 | 673.1 | 194.3 | 39 |
| Freire 2025 | Freire | 194.4 | 34 | 18 | 180 | 42.1 | 16 |
| Friesgaard 2023 | Friesgaard | 1000.2 | 254.16 | 63 | 1038 | 125.89 | 64 |
| Pontes 2021 | Pontes | 741.5 | 150.7 | 68 | 744.3 | 155.4 | 69 |

**H.** Emergence time.

| study | author | mean_control | sd_control | n_control | mean_inter | sd_inter | n_inter |
| --- | --- | --- | --- | --- | --- | --- | --- |
| Arriaza 2021 | Arriaza | 11.8 | 2.5 | 39 | 10.9 | 2.54 | 39 |
| Pontes 2021 | Pontes | 11.26 | 4.94 | 68 | 8.81 | 3.01 | 69 |
| Simoni 2009 | Simoni | 5.1 | 2.7 | 42 | 7.6 | 4.1 | 42 |

**I.** PACU length of stay.

| study | author | mean_control | sd_control | n_control | mean_inter | sd_inter | n_inter |
| --- | --- | --- | --- | --- | --- | --- | --- |
| Green 2024 | Green | 70.94 | 25.83 | 35 | 64.21 | 23 | 29 |
| Moro 2019 | Moro | 70 | 15.54 | 31 | 68.56 | 15.54 | 31 |
| Pontes 2021 | Pontes | 81 | 29.48 | 68 | 56 | 15.42 | 69 |

**J.** Pain at PACU arrival.

| study | author | mean_control | sd_control | n_control | mean_inter | sd_inter | n_inter |
| --- | --- | --- | --- | --- | --- | --- | --- |
| Arriaza 2021 | Arriaza | 2.08 | 2.6 | 39 | 0.49 | 1 | 39 |
| Freire 2025 | Freire | 7.7 | 2.2 | 18 | 0.6 | 1.3 | 16 |
| Green 2024 | Green | 4.5 | 1.85 | 35 | 3.5 | 1.89 | 29 |
| Moro 2019 | Moro | 2.1 | 1 | 31 | 1.8 | 0.9 | 31 |

**K.** Pain scores at 24 hours.

| study | author | mean_control | sd_control | n_control | mean_inter | sd_inter | n_inter |
| --- | --- | --- | --- | --- | --- | --- | --- |
| Arriaza 2021 | Arriaza | 1.2 | 1.28 | 39 | 0.46 | 0.82 | 39 |
| Freire 2025 | Freire | 3.8 | 2.5 | 18 | 1.3 | 2.4 | 16 |
| Friesgaard 2023 | Friesgaard | 2.53 | 2.08 | 63 | 3.44 | 2.08 | 64 |
| Moro 2019 | Moro | 0.35 | 0.77 | 31 | 0.35 | 0.77 | 31 |

**L.** Patient satisfaction.

| study | author | mean_control | sd_control | n_control | mean_inter | sd_inter | n_inter |
| --- | --- | --- | --- | --- | --- | --- | --- |
| Friesgaard 2023 | Friesgaard | 9 | 1.51 | 63 | 8.64 | 2.27 | 64 |
| Moro 2019 | Moro | 191.86 | 7.77 | 31 | 191.93 | 6.99 | 31 |
| Pontes 2021 | Pontes | 178.47 | 121.98 | 68 | 192.71 | 6.41 | 69 |

**M.** Postoperative nausea and vomiting.

| study | author | events_control | n_control | events_inter | n_inter |
| --- | --- | --- | --- | --- | --- |
| Arriaza 2021 | Arriaza | 9 | 39 | 6 | 39 |
| Freire 2025 | Freire | 11 | 18 | 7 | 16 |
| SImoni 2009 | Simoni | 7 | 42 | 4 | 42 |

**N.** Respiratory depression.

| study | author | events_control | n_control | events_inter | n_inter |
| --- | --- | --- | --- | --- | --- |
| Freire 2025 | Freire | 0 | 18 | 0 | 16 |
| Green 2024 | Green | 11 | 35 | 14 | 29 |

**Supplementary Table 6** Opioid Conversions Table.

Opioid doses reported in the included studies were converted to oral morphine equivalents using the ClinCalc online opioid conversion calculator. For each data point, the reported opioid, administration route, and dose were entered into the calculator, and the resulting oral morphine equivalent was recorded in the meta-analysis dataset. The calculator was accessed in July 2025. Medians and interquartile ranges were converted to mean and standard deviations using Wan's method (website attached when needed). No independent conversion formula or modification of the calculator output was applied by the authors.

| **Study** | **Original Opioid, Dose, Unit, and Administration Route** | **Converted Opioid, Dose, Unit, and Administration Route** | **Conversion Source** |
| --- | --- | --- | --- |
| Arriaza 2021 | **Cumulative Opioid Use in 24 h:**  Methadone: 0.74 ± 0.82 mg IV morphine  Control: 1.33 ± 1.24 mg IV morphine  **Intraoperative opioid consumption**  Methadone: 653.4 ± 164.6 mcg remifentanil IV  Control: 624.5 ± 184.5 mcg remifentanil IV | **Cumulative Opioid Use in 24 h:**  Methadone: 1.85 ± 2.05 mg OME  Control: 3.32 ± 3.1 mg OME  **Intraoperative opioid consumption**  Methadone: 163.35 ± 41.15 mg OME  Control: 156.12 ± 46.12 mg OME | https://clincalc.com/opioids/ |
| Freire 2025 | **Cumulative Opioid Use in 24 h:**  Methadone: 2.4 ± 2.5 mg IV morphine  Control: 3.4 ± 3.0 mg IV morphine  **Intraoperative opioid consumption**  Methadone: 43.1 ± 22.7 mcg sufentanil IV  Control: 38.6 ± 20.9 mcg sufentanil IV | **Cumulative Opioid Use in 24 h:**  Methadone: 6 ± 6.25 mg OME  Control: 8.5 ± 7.5 mg OME  **Intraoperative opioid consumption**  Methadone: 53.87 ± 28.38 mg OME  Control: 48.25 ± 26.12 mg OME | https://clincalc.com/opioids/ |
| Friesgaard 2023 | **Cumulative Opioid Use in 24 h:**  Methadone: 42 [10-67] mg OME (Median and interquartile range)  Control: 54.5 [31-83] mg OME (Median and interquartile range)  **Intraoperative opioid consumption**  Methadone: 87.2 (78.7 - 95.7) mL remifentanil 50 mcg/mL IV (Median and interquartile range)  Control: 93.7 (85.9-101.5) mL remifentanil 50 mcg/mL IV (Median and interquartile range) | **Cumulative Opioid Use in 24 h:**  Methadone: 39.52 ± 43.23 mg OME  Control: 56.26 ± 39.45 mg OME  **Intraoperative opioid consumption**  Methadone: 1090 ± 161.17 mg OME  Control: 1171.25 ± 147.94 mg OME | https://clincalc.com/opioids/  https://www.math.hkbu.edu.hk/~tongt/papers/median2mean.html |
| Green 2024 | **Intraoperative opioid consumption**  Methadone: 32.54 ± 9.00 mg OME  Control: 46.94 ± 18.00 mg OME | **Intraoperative opioid consumption**  Conversion not required (doses already in OME) | Not applicable |
| Pontes 2021 | **Intraoperative opioid consumption**  Methadone: 1.1 ± 0.4 mg remifentanil IV  Control: 1.1 ± 0.5 mg remifentanil IV | **Intraoperative opioid consumption**  Methadone: 275 ± 100 mg OME  Control: 275 ± 125 mg OME | https://clincalc.com/opioids/ |

OME: Oral morphine equivalents.

**Supplementary Table 7** TSA Methods.

Trial sequential analysis was conducted using a random-effects model with a two-sided type I error of 5% and a type II error of 20%, corresponding to 80% statistical power. Trial sequential monitoring boundaries were constructed using the Lan–DeMets implementation of the O’Brien–Fleming α-spending function. The required information size was adjusted for between-trial heterogeneity using the observed diversity, yielding a diversity-adjusted required information size for each outcome. All analyses were performed using the TSA Software, version 0.9.5.10, Copenhagen Trial Unit, Denmark.

| **Outcome** | **Included studies (n)** | **Included patients (n)** | **Required Sample Size (n)** |
| --- | --- | --- | --- |
| Cumulative opioid consumption at 24 h | 3 | 239 | 461 |
| Hypoxia | 3 | 233 | 329 |
| Pain at 30 minutes | 3 | 269 | 135 |
| Pain at 60 minutes | 4 | 331 | 715 |
| Patients required postoperative opioids | 4 | 324 | 140 |

**Supplementary File 2** R Codes.

install.packages("meta")

install.packages("metafor")

install.packages("readr")

install.packages("readxl")

library(meta)

library(metafor)

library(readr)

library(readxl)

m.outcome<-metabin(events_inter, n_inter, events_control, n_control,

data = dataset,

method = "Inverse",

method.tau = "REML",

sm = "RR",

byvar = type,

studlab = author)

forest(object,

layout="JAMA or Revman",

sortvar=studlab,

pooled.events=TRUE,

leftcols=c("studlab", "event.e", "n.e", "event.c", "n.c", "w.random", "effect", "ci"),

rightcols=FALSE,

just.addcols="right",

random=TRUE,

common=FALSE,

test.overall.random=TRUE,

overall.hetstat=TRUE,

digits=2,

digits.pval=4,

col.square="darkblue", col.square.lines="black",

col.diamond="black", col.diamond.lines="black",

diamond.random=TRUE,

lab.e="Group 1",

lab.c="Group 2",

fs.heading=12,

colgap="6mm",

colgap.forest.left = "7mm",

label.left="Favors Group 1",

label.right="Favors Group 2")

m.outcomesub<-metacont(n_inter, mean_inter, sd_inter, n_control, mean_control, sd_control,

data = dataset,

method.tau = "REML",

sm = "MD",

subgroup = type,

studlab = author)

forest(MAoutcome,

layout="Revman",

sortvar	= studlab,

leftcols	= c("studlab",

"mean.e","sd.e","n.e","mean.c","sd.c","n.c","w.random","effect","ci"),

header	= c("Studies", "Methadone", "Control"),

leftlabs	= c("Study","Mean","SD","Total","Mean","SD","Total","Weight","MD","95% CI"),

header.line	= "below",

rightcols	= FALSE,

just.addcols = "right",

random	= TRUE,

common	= FALSE,

test.overall.random = TRUE,

overall.hetstat	= TRUE,

digits	= 2,

digits.pval	= 4,

col.square	= "darkcyan",

col.square.lines = "black",

col.diamond	= "black",

col.diamond.lines = "black",

diamond.random	= TRUE,

label.e	= "Methadone",

label.c	= "Control",

fs.heading	= 12,

colgap	= "6mm",

colgap.forest.left = "7mm",

xlim	= c(-100, 100),

at	= seq(-100, 100, by = 20),

label.left	= "Favors Methadone",

label.right	= "Favors Control")

funnel(m.Unilat,

studlab=F,

bg="darkred")

metabias(m.Unilat,

method.bias="Egger",

k.min = X) #minimum number of studies

l1o_m.Unilat<-metainf(m.outcome,

pooled="random")

forest(l1o_m.Unilat,

col.square="lightblue",

col.diamond="gray",

xlim=c(0.1, 5),

xlab="Favors Multiport	Favors Uniport",

rightcols = c("effect", "ci", "I2"))
